# Supplementary material for: The effect of carbamazepine on bone structure and strength in control and osteogenesis imperfecta (Col1a2 +/p.G610C ) mice
Source: J Cell Mol Med. 2022 Jun 14;26(14):4021–31. doi: 10.1111/jcmm.17437 (PMC9279589; doi:10.1111/jcmm.17437)
Supplement: Supplementary file 1 — Figure S1 [file JCMM-26-4021-s004.pdf]

Figure S1. Multi-level-thresholding analysis along the femoral metaphysis showing low-, mid- and high-density bone tissue levels from 9 week old male control and *Col1a2* $^{+/p.G610C}$ mice treated with vehicle or carbamazepine (CBZ) for six weeks by micro-computed tomography (µCT). Graphs shown describe genotype-dependent (A) and treatment-dependent (B, C) differences in bone densities. Data from control and *Col1a2* $^{+/p.G610C}$ mice from panel A are shown in panel B in panel C, respectively, with the effect of CBZ. Data are mean ± SEM; n= 5-8 mice/group. The line on top of the graph indicates a significant difference between the groups ($p<0.05$ was considered minimum significance).

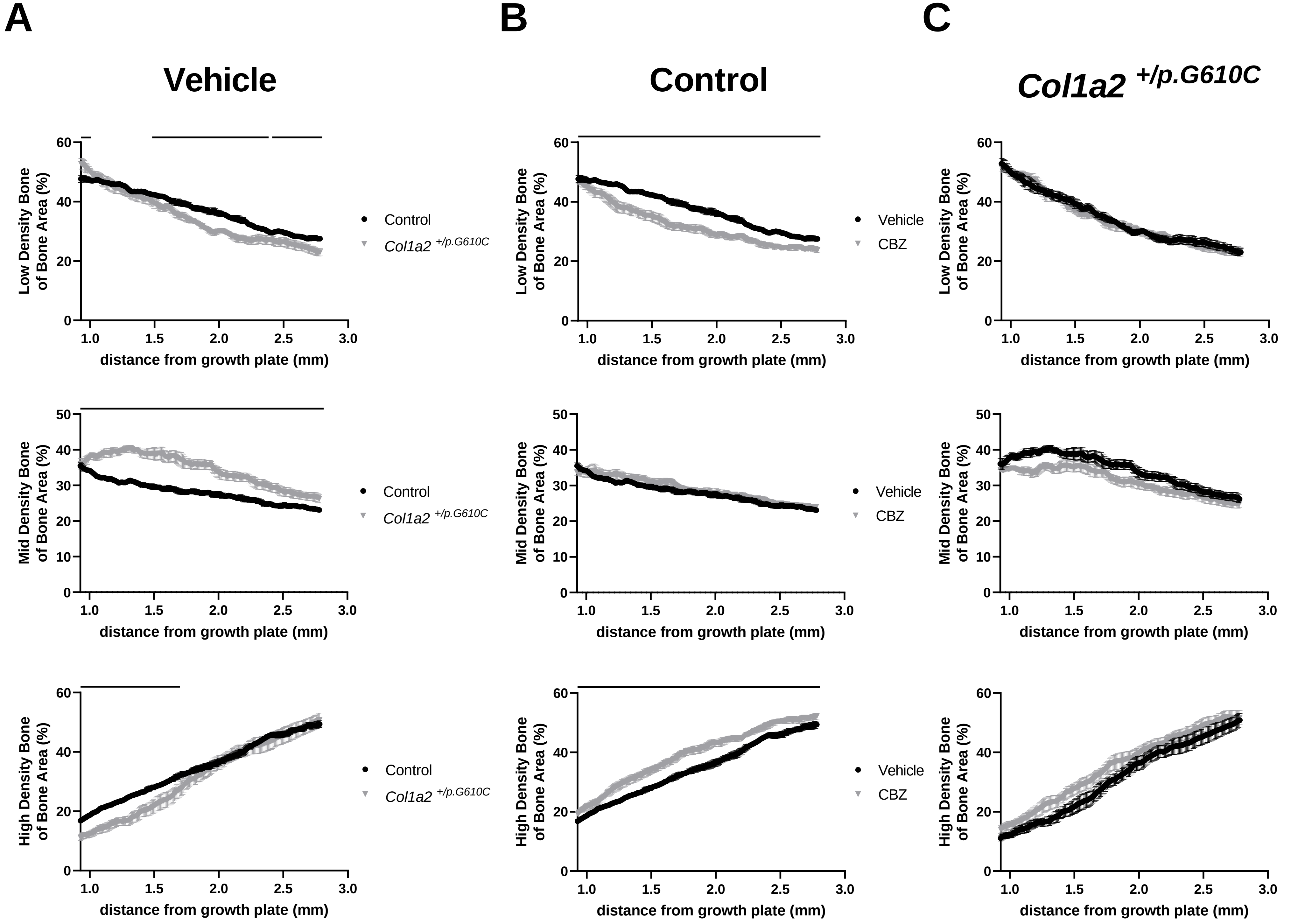
A
B
C
Vehicle
Control
Col1a2 +/p.G610C
Low Density Bone of Bone Area (%)
Mid Density Bone of Bone Area (%)
High Density Bone of Bone Area (%)
distance from growth plate (mm)
Control
Col1a2 +/p.G610C
Vehicle
CBZ
