# Supplementary material for: The effect of carbamazepine on bone structure and strength in control and osteogenesis imperfecta (Col1a2 +/p.G610C ) mice
Source: J Cell Mol Med. 2022 Jun 14;26(14):4021–31. doi: 10.1111/jcmm.17437 (PMC9279589; doi:10.1111/jcmm.17437)
Supplement: Supplementary file 2 — Figure S2 [file JCMM-26-4021-s001.pdf]

Figure S2. Regional analysis of bone composition of tibial cortex from 6 week (A-D) and 9 week (E-H) old male control and *Col1a2*$^{+/p.G610C}$ mice treated with vehicle or carbamazepine (CBZ) for three and six weeks respectively assessed by synchrotron Fourier-transform infrared microspectroscopy (sFTIRM). Ratios were calculated from integrated areas of phosphate (1180-916cm^{-1}), carbonate (890-852cm^{-1}), amide I (1712-1588cm^{-1}) and amide II (1600-1500cm^{-1}) curves. Crystallinity sub-peak was calculated by the integrated area from 1030-1020cm^{-1}. Data shown are mean ± SEM; n= 7-11 mice/group for 6 week old mice and n= 5-8 mice/group for 9 week old mice. + q<0.05, ++ q<0.01 +++ q<0.001 vs. genotype- and treatment-matched region 0 µm. # q<0.05, ## q<0.01, ### q<0.001 vs. treatment- and region-matched controls.

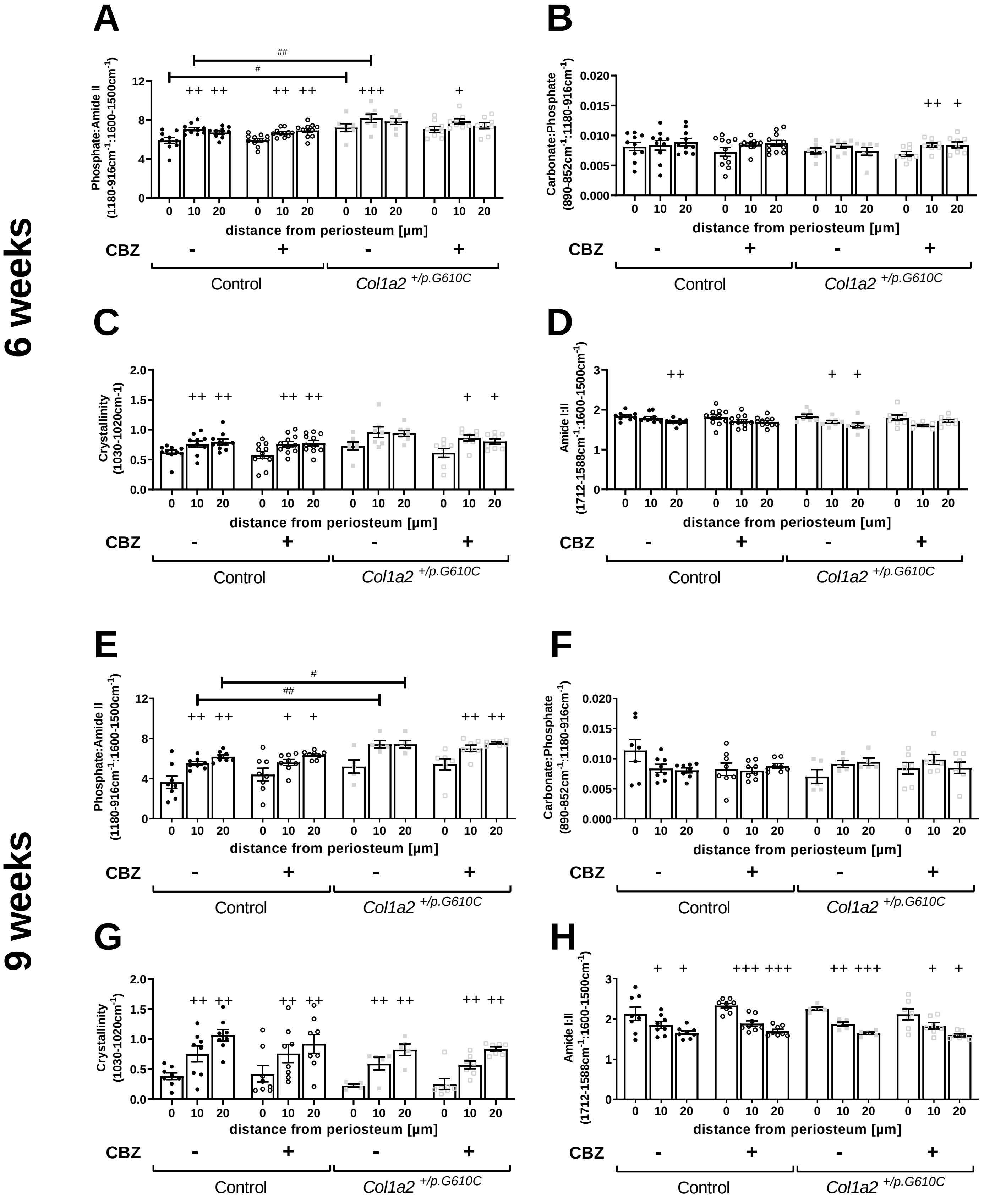
6 weeks
9 weeks
A
Phosphate:Amide II (1180-916cm-1:1600-1500cm-1)
#
##
++ ++ ++ ++ +++ +
distance from periosteum [µm]
CBZ - + - +
Control
Col1a2 +/p.G610C
B
Carbonate:Phosphate (890-852cm-1:1180-916cm-1)
++ +
distance from periosteum [µm]
CBZ - + - +
Control
Col1a2 +/p.G610C
C
Crystallinity (1030-1020cm-1)
++ ++ ++ ++ + +
distance from periosteum [µm]
CBZ - + - +
Control
Col1a2 +/p.G610C
D
Amide I:II (1712-1588cm-1:1600-1500cm-1)
++ + +
distance from periosteum [um]
CBZ - + - +
Control
Col1a2 +/p.G610C
E
Phosphate:Amide II (1180-916cm-1:1600-1500cm-1)
#
##
++ ++ + + ++ ++
distance from periosteum [µm]
CBZ - + - +
Control
Col1a2 +/p.G610C
F
Carbonate:Phosphate (890-852cm-1:1180-916cm-1)
distance from periosteum [µm]
CBZ - + - +
Control
Col1a2 +/p.G610C
G
Crystallinity (1030-1020cm-1)
++ ++ ++ ++ ++ ++ ++ ++
distance from periosteum [µm]
CBZ - + - +
Control
Col1a2 +/p.G610C
H
Amide I:II (1712-1588cm-1:1600-1500cm-1)
+ + +++ +++ ++ +++ + +
distance from periosteum [µm]
CBZ - + - +
Control
Col1a2 +/p.G610C
