# Supplementary material for: The effect of carbamazepine on bone structure and strength in control and osteogenesis imperfecta (Col1a2 +/p.G610C ) mice
Source: J Cell Mol Med. 2022 Jun 14;26(14):4021–31. doi: 10.1111/jcmm.17437 (PMC9279589; doi:10.1111/jcmm.17437)
Supplement: Supplementary file 4 — Table S2 [file JCMM-26-4021-s003.docx]

**Table S2. Additional cortical bone dimensions assessed by µCT and bone strength parameters assessed by three point bending test of femora from 9 week old male control and *Col1a2* *^+/p.G610C^* mice treated with vehicle or carbamazepine (CBZ) for six weeks.** Data shown are mean ± SEM, n= 5-8 mice/group. * p<0.05, ** p<0.01, *** p<0.001 vs. treatment-matched controls. # p<0.05, ## p<0.01, ### p<0.001 vs. genotype-matched controls.

| **Bone dimensions** | | | | |
| --- | --- | --- | --- | --- |
|  | **Control** | | ***Col1a2 ^+/p.G610C^*** | |
|  | Vehicle  (n= 10) | CBZ  (n= 11) | Vehicle  (n= 7) | CBZ  (n= 8) |
| Marrow Area (mm^2^) | 1.07 ± 0.03 | 1.07 ± 0.02 | 0.88 ± 0.02 *** | 0.91 ± 0.02 |
| Mean Polar Moment of Inertia (mm^4^) | 0.34 ± 0.03 | 0.28 ± 0.01 | 0.22 ± 0.02 *** | 0.21 ± 0.01 |
| Medio-lateral width (mm) | 1.72 ± 0.03 | 1.66 ± 0.02 | 1.53 ± 0.03 *** | 1.55 ± 0.02 |
| Cranio-caudal width (mm) | 1.30 ± 0.02 | 1.28 ± 0.01 | 1.23 ± 0.03 * | 1.20 ± 0.02 |
|  |  |  |  |  |
| **Bone strength parameters** | | | | |
|  | **Control** | | ***Col1a2 ^+/p.G610C^*** | |
|  | Vehicle  (n= 11) | CBZ  (n= 16) | Vehicle  (n= 7) | CBZ  (n= 7) |
| **Structural properties** | | | | |
| Yield Load (N) | 8.09± 0.59 | 6.75 ± 0.26 | 6.01 ± 0.72 * | 5.29 ± 0.47 |
| Yield Displacement (mm) | 0.20 ± 0.02 | 0.18 ± 0.02 | 0.11 ± 0.01 * | 0.14 ± 0.02 |
| Stiffness (N/mm) | 45.9 ± 3.67 | 44.0 ± 4.60 | 51.2 ± 5.24 | 34.9 ± 2.92 |
|  |  |  |  |  |
| **Material properties** | | | | |
| Yield Stress (MPa) | 18.5 ± 1.12 | 19.6 ± 1.03 | 21.9 ±1.02 | 20.2 ± 0.95 |
| Yield Strain (%) | 4.29 ± 0.43 | 3.77 ± 0.38 | 2.33 ± 0.28 ** | 2.757 ± 0.33 |
| Elastic Modulus (MPa) | 467 ± 49.3 | 601 ± 77.9 | 936 ± 116 ** | 700 ± 86.2 |
